# Knowing and being known: Psychedelic–assisted psychotherapy and the sense of authenticity

**DOI:** 10.3389/fpsyt.2022.933495

**Published:** 2022-09-20

**Authors:** Lawrence G. Fischman

**Affiliations:** Department of Psychiatry, School of Medicine, Tufts University, Boston, MA, United States

**Keywords:** authenticity, implicit relational knowing, transitional phenomena, partners in thought, noetic feeling, MDMA- and psychedelic-assisted psychotherapy, intersubjectivity, omnipotence

## Abstract

Participants in MDMA- and psychedelic-assisted psychotherapy often emerge from these treatments with new beliefs about themselves and the world. Studies have linked changed beliefs with mystical experiences reported by some participants during drug sessions. While there has been some debate about the epistemic value of drug-induced mystical experiences, and about the need for consent to treatments that may alter metaphysical beliefs, less attention has been given to the sense of authenticity that attends these experiences. In this paper, I consider the intersubjective context in which these changed beliefs arise. I suggest that the sense of authenticity people experience with MDMA- and psychedelic-assisted psychotherapy derives from a simultaneous feeling of knowing and being known. The medications used in these treatments reduce the defensive barriers which ordinarily prevent powerful feelings from being intersubjectively shared, allowing the subject to experience knowing and being known with the therapist and/or internalized or imagined others. In explaining this thesis, I discuss Ratcliffe's “existential feeling;” ipseity in incipient psychosis and psychedelic states; Winnicott's notions of the True Self, omnipotence, creativity, and transitional phenomena; implicit relational knowing and moments of meeting; infant-mother dyad research; predictive processing and the relaxed beliefs model of psychedelic action; the role of the “partner in thought” in knowing and feeling known. I propose that a “transitional space” model of MDMA- and psychedelic-assisted psychotherapy is well-suited for working through “not-me” or dissociated experience

## Introduction

People who use psychedelic drugs often report a noetic sense about the insight or knowledge they gain through their experiences with the drug. The noetic sense is considered a hallmark of both mystical and psychedelic experience. William James's (1) elegant account of the noetic feeling cuts directly to many of the issues raised about these experiences.

Although so similar to states of feeling, mystical states seem to those who experience them to be also states of knowledge. They are states of insight into depths of truth unplumbed by the discursive intellect. They are illuminations, revelations, full of significance and importance, all inarticulate though they remain; and as a rule they carry with them a curious sense of authority for after—time (p. 380).

Walter Pahnke's landmark (2) study of the mystical state induced by psilocybin launched two temporally separated generations of research into the nature of mystical experiences induced by psychedelic drugs. The twin legacy of this research is both the current enthusiasm, if not craze, to apply these drugs to the treatment of a rapidly expanding list of psychiatric diagnoses, as well as the emergence of voices cautioning against accepting mystical experiences as explanations for biomedical treatments (3, 4). Altered metaphysical beliefs arising from mystical states not only pose issues about consent, but raise valid concerns about the epistemic value of insight gained through these experiences (5).

Recent trends have raised the stakes in the debate about the authenticity of these beliefs. Advances in neuroscience, particularly neuroimaging techniques, have tantalized researchers with the possibility of resolving philosophical and scientific questions about consciousness and mind-brain duality. The widespread dissemination of false information on the Internet and social media has upended political systems, justified military invasion, and complicated effective responses to pandemics, among other things. This background, coupled with the still relatively recent history of psychedelic research being totally suppressed for several decades due to misleading information about the drugs' pernicious effects, lends a sense of urgency to settling questions about the authenticity of psychedelic “insight” (6, 7).

Letheby (7) suggests that most of the enduring “mystical experience” benefits of psychedelic treatment (8) could easily fall under “naturalistic” headings, such as changes in one's sense of self, enhanced feelings of unity and connectivity, new psychological insights and existential reflections, and deeply felt emotions [see also (9)]. Beyond simply replacing the fraught term “mystical” with its religious overtones with the less objectionable (to naturalists) term “spiritual,” Letheby maintains that all of these experiences can be derived from changes in self-representation under the “predictive self-binding” model (10). This elegant hypothesis combines predictive processing theory and Carhart-Harris and Friston's (11) “REBUS” model with Sui and Humphreys's (12) “self-binding” theory. In Letheby's account, psychedelics “unbind” or dis-integrate hierarchically supraordinate models of self, which evidence suggests may be generated within the salience (13) and default mode (14) networks in order to efficiently process otherwise overwhelming sensory information. This frees attention from considerations of self-relevance and enables reflection upon experience from outside the perspective of self, similar to the manner advocated within mindful meditation practices.

This explanation, which is essentially a more nuanced view of the process that is elsewhere commonly referred to as ego dissolution, accounts well for much of the phenomenology of psychedelic experience. However, it does not specifically address the relational context of authenticity and knowing. Erickson (15) writes, “In emphasizing the importance of relationships, the postmodern challenge to authenticity becomes one of context. It is no longer a question of being “true to self” for all time, but rather of being true to self-in-context or true to self-in-relationship” (p. 139). Experimental evidence suggests that “people largely do not feel authentic (or inauthentic) unless another is present” (16).

In the following, I will try to address the intersubjective dynamics of experiencing a kind of knowing (17) that feels “realer than real.” Why do noetic feelings occur? Why do noetic feelings occur in psychedelic states? What gives them a sense of authority and authenticity? Why do they awaken a desire to live more authentically afterwards? Are noetic feelings truly unmediated (18), or is a “shadow” mediator present?

I suggest that observations drawn from infant-caregiver dyad research and psychoanalytic practice can enrich this account. The intertwined dynamics of “knowing and being known” help explain the noetic sense people have about psychedelically-experienced insight as well as the sense of authenticity which often accompanies, and is promoted by, this process. This attempt to clarify the origins and depth of the noetic feeling does not engage the debate about the epistemic value of certain metaphysical insights attained in a psychedelic state, which may be subjectively experienced as mystical or even sacred. The observations I draw upon continue a naturalistic account of psychedelic experience while remaining initially agnostic with respect to the non-naturalistic metaphysical contents of some subjective experiences (19, 20), allowing these to unfold naturalistically, as it were, in the crucible of subsequent psychedelic and non-psychedelic experience.

Situating psychedelic-assisted psychotherapy within models derived from psychoanalytic practice, infant observation, and predictive processing theory (incorporated in Letheby's predictive self-binding hypothesis above) may help shift the direction of research with these agents away from controversial assessments of effectiveness based on parameters measured on the Mystical Experiences Questionnaire [MEQ; (21)] and the Hood Mysticism Scale (22) toward parameters that measure the benefits of psychedelically attained insight (23) along existential dimensions such as meaningfulness, connectedness, reductions in defensiveness, acceptance and what I will focus upon here, authenticity.

Such a shift in focus comes quite naturally from studies which have examined the benefits of psychedelic-assisted psychotherapy in treating existential anxiety in subjects with terminal illness. While some authors (24) suggest mystical or transpersonal encounters as the reason some subjects have experienced dramatically reduced anxiety and increased acceptance, first person accounts from these studies also lend themselves to more naturalistic interpretations as suggested above by Letheby. Such accounts have led Letheby (7) to conclude his philosophical treatise on psychedelics by following Grob in viewing psychedelics as “existential medicines” (p. 24).

## Authenticity and existential feelings

MDMA-assisted psychotherapy has been associated with increased feelings of authenticity (25). “Authenticity” is the term we often use to describe our sense of how real something feels. Ratcliffe (26) proposes that one's sense of reality about things is an “existential feeling.” Existential feelings are the ways we “already *find ourselves in the world*” (p. 2). They are “inextricable from our *sense of reality*” (p. 2). He clarifies, “They are not intentional states, directed at however many objects, and they are not feelings of the body or some part of it. Instead, they amount to a felt sense of *belonging* to the world” (p. 2; emphasis added). Ratcliffe says that alterations in one's felt way of belonging to the world affect one's sense of reality.

People sometimes talk of feeling alive, dead, distant, detached, dislodged, estranged, isolated, otherworldly, indifferent to everything, overwhelmed, suffocated, cut off, lost, disconnected, out of sorts, not oneself, out of touch with things, out of it, not quite with it, separate, in harmony with things, at peace with things or part of things. There are references to feelings of unreality, heightened existence, surreality, familiarity, unfamiliarity, strangeness, isolation, emptiness, belonging, being at home in the world, being at one with things, significance, insignificance, and the list goes on. People also sometimes report that “things just don't feel right”, “I'm not with it today”, “I just feel a bit removed from it all at the moment”, “I feel out of it” or “it feels strange” (p. 2).

These descriptions are examples of how one's sense of reality is determined by the way one feels one belongs the world.

Before discussing the clinical implications of the connection between one's sense of reality and knowing, I will mention a few more salient points Ratcliffe makes about existential feelings. Existential feelings structure the way one perceives the world. They shape *the way that things matter, feel significant*. “The horizonal structure of experience incorporates not only relations of practical and perceptual accessibility but also ways in which things appear significant to us” (p. 8). Also, “existential feelings are associated principally with the kinds of mattering that experience incorporates” (p. 10). He emphasizes that existential feelings are concerned with *the kinds of possibilities* we feel are open to us. “Once we allow that experience incorporates various kinds of possibility, it becomes possible to explain how something could look exactly the same as it previously did and yet very different. The perceived properties remain intact but the kinds of experienced possibility habitually associated with them have changed” (p. 12). In other words, one's existential feeling at the moment of perception determines whether it is felt as an ordinary, already known, largely insignificant observation or as meaningful in a new way. This has important implications for insight in psychotherapy, including psychedelic-assisted psychotherapy. With psychedelics, something which someone has long known can suddenly strike one with new significance, can be experienced as powerfully meaningful insight (27).

Ratcliffe also points out that “existential feelings are not specific to our relationship with the impersonal world; they are also ways of finding ourselves with other people” (p. 5). They determine the kinds of possible experiences we can have with people. This connects existential feelings, or one's sense of reality, with interpersonal expectancies, about which we will have a good deal more to say below.

Finally, Ratcliffe mentions that in addition to involving ways we find ourselves in relation to other people and the impersonal world, “existential feelings involve experience of the body” (p. 20). This bears directly upon the altered sense of reality or “knowing” one may experience in psychosis and psychedelic states, as I will discuss below. By altering the way we know, including the way we experience or know our bodies, psychedelics alter our sense of reality, our way of belonging to the world. Ratcliffe's discussion about how existential feelings shape the way things matter to us, the way we determine what our possibilities are, and the way we belong within our bodies and with other people, provides a useful way of thinking about the connection between the way we know things and our sense of reality, or authenticity.

## Ipseity and the sense of the real

In using a phenomenological approach emphasizing the “lived experience” of schizophrenia Sass and Parnas (28) have identified an alteration in “ipseity” as the fundamental underlying disturbance in the disorder. “Ipseity refers to the experiential sense of being a vital and self-coinciding *subject* of experience or first *person perspective on the world*” (p. 428). Changes in ipseity present in two complementary forms: hyperreflexivity, “an inflated self-consciousness and heightened awareness of aspects of one's experience that are normally tacit and implicit and “in the background” of experience (e.g., awareness of the act of breathing or sensations while walking, or of the “inner speech” that mediates our thinking” [(29), p. 13]; and diminished self-affection, which “emphasizes a complementary aspect of this very same process— the fact that what once was tacit is no longer being inhabited as a medium of taken-for-granted selfhood” [(27), p. 430]. These changes in the way one experiences one's self and one's body are virtually identical to the changes in experiencing one's self and body reported by subjects with psychedelics (30–36).^1^ With both psychedelics and the onset of schizophrenia, ipseity is disrupted. Kinesthetic sensations, proprioception, walking, talking, breathing, swallowing, etc., which normally take place automatically, outside of awareness, become a focus of attention.

When ipseity is disturbed, “there is necessarily a disruption of a person's “grip” or “hold” on the conceptual or perceptual field of awareness” [(29), p. 14]. This alters the way we “are always *in relationship to* the world/others” [(29), p. 14], the way that things matter to us. The main point here is to illustrate that these drugs, in altering the way we know or recognize our selves (disrupting ipseity), influence our pre-reflective way of being in the world, our sense of reality.

## Authenticity and knowing

A brief anecdote may illustrate the connection between “knowing,” which I will argue is central to one's sense of reality, and the sense of “belonging in the world.” Recently, I attended a family event at which I was introduced by my cousin and her husband to their 9 year old son. After briefly scanning my appearance, he consulted a loose-leaf binder he was carrying and matter-of-factly reported, “I have no record of you.” I reacted with amusement to this candid assessment and assured his slightly horrified parents that I was not offended. The implication of the story, as I later recounted humorously, was that because my young cousin had no record of me, I did not exist. To my young cousin I was, in today's parlance, a non-starter. But as I started writing this paper and thinking about Ratcliffe's existential feelings, I saw new value in my young cousin's succinct greeting. His binder was his way of knowing, his trusted source; a record of people whom he knew, people whose world he belonged to. Since he had no record of me, I did not belong in his world. In his precocious and direct way, he made explicit the connection between ways of knowing and a sense of belonging in the world.

Existential feelings are “pre-intentional,” in that they “determine what kinds of intentional state are amongst one's possibilities” [(26), p. 10]. In order to determine what kind of intentional states are possible, therefore, one must examine how one's world of possibilities is constructed. My basic premise is that authenticity is a *feeling that involves shared knowledge*. The experience of authenticity occurs privately, individually, but the object of the feeling has an intersubjective context, which can be described as a simultaneous sense of “knowing and being known.” I will argue that it is in the intersubjective world of the infant-caregiver dyad that this feeling takes shape, where the roots of one's sense of reality and the feeling of authenticity lie. The shared experience of knowing and being known is extended in various settings, including Winnicott's (37) transitional phenomena, the psychotherapeutic setting, and in psychedelic states.

## The true self and the sense of the real

In the psychedelic literature, the sense of authenticity, the feeling that something is “realer than real” is often applied to beliefs that strike the outside observer as an illusion. This paradoxical association makes some sense when considered in the light of Winnicott's ideas about the sense of the real, which reverberate throughout his discussion of the “True Self” and transitional phenomena.

For Winnicott (38), feeling real is a manifestation of the True Self. “At the earliest stage the True Self is the theoretical position from which come the spontaneous gesture and the personal idea. The spontaneous gesture is the True Self in action. Only the True Self can be creative and only the True Self can feel real. Whereas, a True Self feels real, the existence of a False Self results in a feeling unreal or a sense of futility” (p. 147). Winnicott explains (more or less): “Periodically the infant's gesture gives expression to a spontaneous impulse; the source of the gesture is the True Self, and the gesture indicates the existence of a potential True Self. We need to examine the way the mother meets this infantile omnipotence revealed in a gesture (or a sensori-motor grouping)” (p. 144).

In this packed statement, Winnicott tells us that the True Self is a “potential,” is the source of the “spontaneous gesture,” and that this gesture manifests the infant's “omnipotence.” It is in the way that the mother responds to the spontaneous gesture that the fate of the potential true self lies. “The good-enough mother meets the omnipotence of the infant and to some extent makes sense of it. She does this repeatedly. A True Self begins to have life, through the strength given to the infant's weak ego by the mother's implementation of the infant's omnipotent expressions” (p. 144). Failure to “implement” the infant's omnipotence leads to compliance on the part of the infant, and the beginnings of the False Self. “It is an essential part of my theory that the True Self does not become a living reality except as a result of the mother's repeated success in meeting the infant's spontaneous gesture or sensory hallucination” (p. 144).

The two paths of development, True Self and False Self, may thus be traced to the mother's contingent response to what Winnicott pointedly terms a sensory hallucination, or illusion. In the case of the mother's empathic (knowing) recognition of the infant's need, the “infant begins to believe in external reality which appears and behaves as by magic (because of the mother's relatively successful adaptation to the infant's gestures and needs), and which acts in a way that does not clash with the infant's omnipotence. On this basis the infant can gradually abrogate omnipotence” (p. 145). In other words, by meeting the infant's need in close temporal proximity to the infant's experiencing of the need, the mother “implements” the infant's illusion of omnipotence: “you appeared *because* I wished for you.” Somewhat paradoxically, enabling the infant's omnipotence allows the infant to abrogate omnipotence, because the infant is given leeway to discover the illusory nature of omnipotence on his/her own.

What is especially relevant in this to understanding the origins of the sense of the real is the phrase “the infant begins to believe in external reality which appears and behaves as by magic.” *Belief*, a sine qua non dimension of the sense of the real, *arises from illusion*, an illusion granted by the good enough mother who senses the infant's need, *including the need for belief*. Intuitively, the mother promotes the infant's confidence in his/her ability to control (at least some aspects of) the objects in his/her environment.

Winnicott (39) summarizes this earliest form of object relations:

The initiation of object-relating is complex. It cannot take place except by the environmental provision of object-presenting, done in such a way that the baby creates the object. The pattern is thus: the baby develops a vague expectation that has origin in an unformulated need. The adaptive mother presents an object or a manipulation that meets the baby's needs, and so the baby begins to need just that which the mother presents. In this way the baby comes to feel confident in being able to create objects and to create the actual world. The mother gives the baby a brief period in which omnipotence is a matter of experience (p. 61).

The infant's experience of the “subjective object” (see quote below) affords him the same sense of control over and connection to the object as he has to himself. If not exactly a sense of certainty, the subjective experience of the mother's reliability leads to a sense of confidence and trust [(40), p. 102], which bears directly upon one's sense of the real, including that which occurs in mystical states.

In fact, Winnicott (41) recognized this connection, too:

In thinking of the psychology of mysticism, it is usual to concentrate on the understanding of the mystic's withdrawal into a personal inner world of sophisticated introjects. Perhaps not enough attention has been paid to the mystic's retreat to a position in which he can communicate secretly with subjective objects and phenomena, the loss of contact with the world of shared reality being counterbalanced by a gain in terms of feeling real (p. 185).

Thus, the sense of the real, or authenticity, can be traced to the earliest way of relating to objects, which Winnicott termed the “subjective object,” in which the foundations of confidence and belief arise from the mother's “implementation” of the infant's “illusion” of omnipotence. This enables the True Self to develop, in which the sense of the real lives. Where the holding environment (mother) has not afforded this adaptation, various forms of psychopathology arise, many of which revolve around a False Self (42) which may sometimes be corrected in long, painstaking, dynamically based psychotherapies with a patient analyst who is willing to wait indefinitely for the patient to discover that which is waiting to be found [(43), p.37]. Psychedelics, by enabling a mode of experiencing which affords contact with the subjective object, offer promise of accelerating this discovery.

## Transitional phenomena, creativity, and the sense of the real

Winnicott (44) states, “From birth … the human being is concerned with the problem of the relationship between what is objectively perceived and what is subjectively conceived of” (p. 11). This is precisely the issue (an illusion or not?) which causes controversy in psychedelic-assisted psychotherapy. His seminal concept of transitional phenomena describes how the illusion-disillusionment process applies to this problem. This concept is given in the statement: “*Of the transitional object it can be said that it is a matter of agreement between us and the baby that we will never ask the question: ‘Did you conceive of this or was it presented to you from without?* ’ *The important point is that no decision on this point is expected. The question is not to be formulated*” (p.12). In applying the same “matter of agreement” to these treatments, psychedelic-assisted psychotherapy may be considered a transitional phenomenon. It is made a transitional phenomenon by the way we observe it: “an essential feature of transitional phenomena and objects is a quality in our attitude when we observe them” [(45), p. 96].

In elaborating his concept of transitional phenomena, Winnicott extends his notion, discussed above in connection with the True Self, of the relation between creativity, illusion, and the sense of the real. He uses the meaning of creativity “that refers to a coloring of the whole attitude to external reality. It is creative apperception more than anything else that makes the individual feel that life is worth living” [(40), p. 65]. The alternative is “a relationship to external reality which is one of compliance…. Compliance carries with it a sense of futility for the individual and is associated with the idea that nothing matters and that life is not worth living” [(39), p. 65]. Here Winnicott equates whether life is worth living with contemporary ideas about living authentically derived from humanistic psychology (46, 47). Transitional phenomena allow one to adopt a creative and therefore authentic relationship with reality.

In responding to the infant's spontaneous gesture, the good-enough mother's adaptation allows the infant to experience the illusion of creating the object. But at a certain point in development, just when the biologically determined primary maternal preoccupation begins to wane, the mother senses a shift in the infant's need in the direction of autonomy and changes her adaptation from that of allowing illusion to that of allowing gradual disillusionment. This enables the infant to experience tolerable frustrations with respect to her responsiveness, and ushers in a new way of relating to objects. Winnicott identifies an “intermediate area between the subjective and that which is objectively perceived” [(44), p. 3], a “potential space”^2^ (40) where transitional phenomena occur. Winnicott (44) states:

Transitional objects and transitional phenomena belong to the realm of illusion which is at the basis of initiation of experience. This early stage in development is made possible by the mother's special capacity for making adaptation to the needs of her infant, thus allowing the infant the illusion that what the infant creates really exists (p. 13).

Whereas younger infants in a good-enough holding environment experience the (subjective) object as *created*, older infants paradoxically experience the (transitional) object as both *created and found*.

In health the infant creates what is in fact lying around waiting to be found. But in health the object is created, not found…. A good object is no good to the infant unless created by the infant. Shall I say, created out of need? Yet the object must be found in order to be created. This has to be accepted as a paradox… [(41), p. 181].

The paradox of (feeling that one is) creating that which is found (already exists in external reality) confers a sense of authenticity to transitional phenomena. Were the object simply created and not found it would have a hallucinatory, dream-like quality; were the object found but not created, it would lack relevance to oneself. The sense of the real derived from the simultaneous experience of created and found occurs within transitional phenomena because the subject is not forced to decide between the two. The same matter of agreement obtains in play in general, and the entire sphere of cultural activity throughout the lifespan, all of which can be viewed, therefore, as transitional phenomena.

This intermediate area of experience, unchallenged in respect of its belonging to inner or external (shared) reality, constitutes the greater part of the infant's experience, and throughout life is retained in the intense experiencing that belongs to the arts and to religion and to imaginative living, and to creative scientific work [(44), p. 13].

These areas of “intense experiencing” are those which people often regard as most personally meaningful, activities in which individuals make contact with their “real” feelings, ideals, and values. This makes sense when considering creativity's roots, which, in Winnicottt's view, may be traced to the True Self and to the infant's illusion of omnipotence and the gradual process of disillusionment. Importantly, this same path is traced by the sensitive psychotherapist who recognizes this “intermediate area of experience” as *a process of experiencing;* that is, an approach to “the truth,” or the sense of the real through gradual disillusionment over time. Just as “a good object is no good to the infant unless created by the infant,” so a clever interpretation is no good to a patient unless created by the patient. As Winnicott (43) puts it:

The patient is not helped if the analyst says: “Your mother was not good enough” … “your father really seduced you” … “your aunt dropped you.” Changes come in an analysis when the traumatic factors enter the psycho-analytic material in the patient's own way, and within the patient's omnipotence. The interpretations that are alterative are those that can be made in terms of projection. The same applies to the benign factors, factors that led to satisfaction. Everything is interpreted in terms of the individual's love and ambivalence. The analyst is prepared to wait a long time to be in a position to do exactly this kind of work (p. 36).

Winnicott's case illustrations are replete with cautionary notes to the reader about how critical it is to withhold interpretation until the moment it can be created, so to speak, by the patient. He came to this relatively late in his practice, even expressed regret at how much deep change in patients he foreclosed by his need to interpret prematurely [(40), p. 86]. By waiting, the analyst treats interpretation in parallel with the infant's mother, who “places the actual breast just there where the infant is ready to create, and at the right moment” [(44), p. 11]. In this statement, Winnicott refers not only to the mother's feeding the infant at the moment of hunger, but her general adaptation to the infant's needs, which enables the infant to feel that mother's responsiveness “is, as it were, under the baby's magical control” [(44), p. 11].

This process relates directly to the connection between intersubjective knowing and being known and the sense of authenticity. It is more easily recognized in the treatment situation, in which the patient feels known on a deep level when she *finds* in the analyst's response to her experience an interpretation which coincides with that which she was in the process of formulating or *creating* herself. This constitutes a potential “moment of meeting,” as discussed below. In anticipation of this discussion, I must suggest some modifications to Winnicott's theorizing about omnipotence, illusion, creating and finding that may make the moment of meeting, of knowing and being known, more understandable. These modifications are intended to apply to the discussion above as well, which will allow this discussion to be re-read with a different emphasis.^3^

I suggest that much of the time the infant's sense of control over the object is not an illusion, that the microanalysis of video recordings of mother-infant interaction reveals the bidirectional regulation of their social interactions, and shows the remarkable range of expression, gesture, movement, vocalization, etc, with which the infant can regulate the mother's behavior (48). I also suggest (what intuitively makes more sense in view of the asymmetrical power relationship between the two), that any sense of omnipotence the infant experiences at this early stage is primarily associated with the mother, and is based on the infant's sense of the remarkable physical and mental capacities of the mother, including her capacity to sense the infant's need. This may be the primary way in which omnipotence plays a role in early infant experience, as an assumption that the mother knows what the infant is experiencing {This may account for the observation that during ordinary affect attunement, the infant behaves as though nothing special is taking place. It is only through the infant's registration of surprise (or more) when affect attunement is disrupted that one can infer that the infant was experiencing a sense of being attuned with beforehand [see (49), p. 149, (50), p. 36]}. I also suggest that the process of illusion-disillusionment initiated and facilitated by the mother is primarily directed at the infant's illusion *of the mother*'*s* omnipotence, including her omniscience, her ability to know everything, including the infant's state of mind– *not the infant*'*s* omnipotence.

In his introduction to the book, *Playing and Reality* (37), which also serves as the lead-in to the fourth and final version of his paper, “Transitional Object and Transitional Phenomena” (44), Winnicott states:

I am drawing attention to the *paradox* involved in the use by the infant of what I have called the transitional object. My contribution is to ask for a paradox to be accepted and tolerated and respected, and for it not to be resolved. By flight to split-off intellectual functioning it is possible to resolve the paradox, but the price of this is the loss of the value of the paradox itself.

This paradox, once accepted and tolerated, has value for every human individual who is not only alive and living in this world but who is also capable of being infinitely enriched by exploitation of the cultural link with the past and with the future [(37), p. xii].

The paradox that must be accepted is that “the baby creates the object, but the object was there waiting to be created” [(40), p. 89]. But Winnicott also emphasizes that the crux of his idea about the transitional object is *the way the object is used*, not its nature. It is clear that the creative ways in which the child uses the transitional object are an illusion. It is animated, given life by the infant/child–“it must seem to the infant to give warmth, or to move, or to have texture, or to do something that seems to show it has vitality or reality of its own” [(44), p. 5]. Therefore, I suggest that the illusion-disillusionment process faced by the child who creates the transitional object is not so much about the child's omnipotence (his feeling of having created the object), though it may be partly about that, as it is about the child's knowing and being known by the object. In this view, “*the potential space between the subjective object and the object objectively perceived*” [(45), p. 100] is understood to mean a transitional space between the subjective omnipotent object who knows all of the subject's feelings and needs and the objectively frustrating object who is sometimes distracted, depressed, rigid, hateful, self-involved, or otherwise mis-attuned. The child uses the transitional object creatively as a means of creating and finding potential selves in it, *including finding what the actual object does not know*. All of the value ascribed by Winnicott to the transitional phenomena as an unchallenged area of play and cultural experience, including the value of paradox and, more germane to our topic of authenticity, the feeling of being alive and real, has less to do with preserving and gradually relinquishing the infant's omnipotence than with finding the (true) self in the other, creating an object who knows what the infant experiences, and learning how to deal with one who doesn't. In this view, the adaptation made by the good-enough mother at the beginning of transitional phenomena is triggered by *her identification with the infant beginning to know that the mother both knows and does not know about the infant*'*s experience*.

I suggest that the question that the sensitive parent intuitively knows not to ask is not whether the object was created or found by the child (though this, too, should not be asked), but *whether the object is alive*, (i.e., real), because the parent understands that *it is through the living object that the child feels alive*. Winnicott states that “transitional phenomena are allowable to the infant because of the parents' intuitive recognition of the strain inherent in objective perception, and we do not challenge the infant in regard to subjectivity or objectivity just here where there is the transitional object” [(44), p. 13]. I suggest that much of this strain relates to objectively perceiving the limits of the parent's capacity to know the child, and therefore, the child's capacity to know herself. The child feels alive in the transitional space, the “place where we live”^4^ (51), because the child has created a transitional object in order to know who she is.

In the next section, I adduce theory which helps explain why the infant looks to the object to know who he/she is.

## Knowing and being known

The most basic premise in my argument for the origins of the sense of authenticity relies upon its being intersubjectively constituted. In a footnote to his paper about the True and the False Self, Winnicott quotes himself. “I once said: ‘There is no such thing as an infant’, meaning, of course, that whenever one finds an infant one finds maternal care, and without maternal care there would be no infant” [(43), p. 38]. Of course, he did not intend this in a literal sense, but it is evident in the emphasis he gives this here and elsewhere that he meant this literally in the intersubjective sense, where he takes the position, which is adopted here, that in discussing the infant's development, “It is not possible to state what takes place by reference to the infant alone” [(38), p.144]. One of the implications of “what takes place” is how the infant comes to know her/ himself. Winnicott asks, “What does the baby see when he or she looks at the mother's face? I am suggesting that, ordinarily, what the baby sees is himself or herself. In other words, the mother is looking at the baby and *what she looks like is related to what she sees there*” [(52), p. 112]. Winnicott's precise, italicized use of language reveals his highly specific intention here, which is that on a perceptual level (“*what she looks like*”) the infant feels that the mother's facial expression reveals something that is specifically related to her/ himself.

This places Winnicott's observation alongside current generative theories about the transmission of knowledge, or for our purposes, *knowing*. Gergely and Csibra (53), building upon Russell's (54) recognition of humans' use of ostention to signal communicative intent and Sperber and colleagues (55, 56) theories of relevance and epistemic vigilance, posited a theory of natural pedagogy. In this view, communicating agents use ostensive cues to induce a temporary suspension of epistemic vigilance in their addressees and regard what is to be communicated as personally relevant, generalizable, and shared by others. Fonagy (57) incorporated these concepts into his theory of mentalization and later extended them into the concept of epistemic trust: “The very experience of and having our subjectivity understood—of being mentalized—is a necessary trigger for us to be able to receive and learn from the social knowledge that has the potential to change our perception of ourselves and our social world” [(58), p. 372]. A key component of this formulation is that “the very experience” of feeling understood, of feeling that one's subjective experience is known by another, is the pre-condition for acquiring knowledge about oneself. The connection between knowing and feeling known is here made explicit. By re-stating this formulation in the first person, its implications for my argument here become even clearer. “It is because I truly feel that you understand (know) what I am feeling that I may trust what you are about to tell me enough to allow it to affect the way I think about myself and the world.” This view of mentalizing bears directly on establishing a sense of authenticity in psychotherapy. “Mentalizing in therapy is a generic way of establishing epistemic trust (trust in the authenticity and personal relevance of interpersonally transmitted information)” [(58), p. 372].

Returning now to Winnicott's observation, “the mother is looking at the baby and *what she looks like is related to what she sees there*,” there is an implication of bi-directionality in this. Winnicott implies that “*what she looks like*” is *influenced* by “*what she sees there*.” This interpretation of Winnicott's remark is suggested by his subsequent statements (52). “I can make my point by going straight over to the case of the baby whose mother reflects her own mood or, worse still, the rigidity of her own defenses. In such a case what does the baby see?” (p. 112). Winnicott tells us that in this case, infants “look and they do not see themselves” (p. 112). They see only their mother's face, not a reflection of themselves. When this happens, “perception takes the place of apperception, perception takes the place of that which might have been the beginning of a significant exchange with the world, a two-way process in which self-enrichment alternates with the discovery of meaning in the world of seen things” (p. 113). Infants see themselves in the mother's face only when the mother is attuned to the infant's needs, something that is “naturally done well by mothers who are caring for their babies” (p. 112) thanks to their “primary maternal pre-occupation” (59). This enables self-enrichment and the discovery of meaning, components of the process of knowing.

Winnicott illustrates his ideas about the infant discovering herself in her mother's face by citing examples from psychoanalysis with adults. In one case, from which Winnicott (52) acknowledges he learned much of what he discovered about the importance of infants seeing themselves reflected in their mothers' faces, a female patient whose mother and nurse had both been depressed was in a very long “analysis [which] involved a severe and deep regression to infantile dependence” (p. 115). She was finally able to “come through, late in life, to *feeling real*” (p. 115, emphasis added) after she sent Winnicott, who already had a portrait of the woman's rigid mother, a portrait of the depressed nurse she had when young. Around the same time, she saw a picture of Winnicott's face on a book cover and asked him for an enlarged version of the picture. He sent the picture to the woman along with an interpretation:

What she needed to be told was that my lined face had some features that link for her with the rigidity of the faces of her mother and her nurse. I feel sure that it was important that I knew this about the face, and that I could interpret the patient's search for a face that could reflect herself, and at the same time see that, because of the lines, my face in the picture reproduced some of her mother's rigidity (p. 116).

After he had written his ideas about this case, in another case, a woman “very much concerned with the stage of the establishment of herself as an individual” (p. 115), brought in so much material about trying to see herself reflected in the face of another that it seemed to Winnicott almost as though she had read his paper on the subject. “She referred to a detail in a book about Francis Bacon” (p. 117), whom Winnicott discusses in “Mirror-role of Mother and Family in Child Development” (52), and said “Francis Bacon ‘says that he likes to have glass over his pictures because then when people look at the picture what they see is not just a picture; they might in fact see themselves”’ p. 117). She then discusses Lacan's “Le Stade du Miroir,” which Winnicott also discusses in this paper, though “she was not able to make the link that I feel I am able to make between the mirror and the mother's face” (p. 117).

Winnicott does not interpret this from a sense that it would be premature, and thus annihilate the woman's creativity, which must follow from discovery in keeping with the maturational process (more on this below). But he uses the case to illustrate his ideas about psychoanalysis and its relation the developmental process in general.

This glimpse of the baby's and child's seeing the self in the mother's face, and afterwards in a mirror, gives a way of looking at analysis and at the psychotherapeutic task. Psychotherapy is not making clever and apt interpretations; by and large it is a long-term giving the patient back what the patient brings. It is a complex derivative of the face that reflects what is there to be seen. I like to think of my work this way, and to think that if I do this well enough the patient will find his or her own self, and will be able to exist and to feel real. Feeling real is more than existing; it is finding a way to exist as oneself, and to relate to objects as oneself, and to have a self into which to retreat for relaxation (p. 117).

In this paragraph, Winnicott makes explicit the connection, both in development and in psychoanalysis, between the sense of authenticity, or feeling real, and the discovery of one's self in the shared intersubjective field with another. Within the intersubjective field, the process of knowing oneself and feeling known by the other feel real and coincide.

## Implicit relational knowing and authenticity

Winnicott's remark about what the baby sees when she looks into her mother's face anticipated much of the fertile research and theorizing that has come from video microanalysis of mother-infant dyads, and in particular, theories about pre-reflective, implicit relational knowing and moments of meeting. Implicit relational knowing, the “how ‘to be with’ someone” [(60), p. 905], is based on the “fittedness” between the subjectivities of two individuals. By recalling Ratcliffe's definition of existential feelings as pre-reflective ways of belonging in the world, implicit relational knowing, which concerns procedural, pre-reflective knowledge about being with others, may be thought of as a specific form of existential feeling related to *belonging with another person*. An example of the infant's procedural knowing is “what form of affectionate approaches the parent will welcome or turn away” [(60), p. 905]. It offers a rationale for Winnicott's insistence on the importance of accepting the paradoxical experience of something being simultaneously created and found, and how this relates to the sense of authenticity. Implicit relational knowing also highlights the importance of viewing transitional phenomena with the slight modification suggested above, as a place where the child creatively adapts to relinquishing the omnipotent object.

The theory of implicit relational knowing (60–62) was developed by clinician researchers and theorists with a combined interest in infant observational data and psychoanalytic theory to shed light on both areas of study, with the ultimate aim of explaining “the *something more* than interpretation” [(60), p. 903] which accounts for the process of change in psychoanalytic treatment. “Our attention was drawn to the observation that most patients remember “special moments” of authentic person-to-person connection with their therapists, moments that altered their relationship with him or her and thereby their sense of themselves” [(62), p. 284]. The “something more” that implicit relational knowing grapples with includes “notions such as ‘moments of meeting’, the ‘real’ relationship, and authenticity” [(60), p. 903]. In psychoanalytic treatment, a “moment of meeting” is an event “that rearranges *implicit relational knowing* for patient and analyst alike” [(60), p. 906]. Patients tend not to recall specific interpretations, but rather a sense of being with, of simultaneously knowing and being known by their therapists [(63), p. 713].

Following Sander (64), the process of knowing and being known is construed as both an ongoing and an acute “recognition process” (p. 589) in which the intentions of one subjectivity are recognized in another's, and recognized by both to be co-occurring, or co-created. The recognition process is theorized as an adaptation requiring a specific fittedness between two biological systems or organizations, that through their intersection, following non-linear dynamic systems theory (65, 66), lend greater coherence and complexity to each, a process experienced subjectively as “vitalizing” or feeling whole [(50), p. 34]. This greater coherence and complexity is what Tronick (67, 68) called a “dyadic expansion of consciousness.” Within an ongoing framework of recurrent, adaptive, regulatory interaction between infant and mother, or analysand and analyst, a “key moment of specificity” arises which becomes “the now moment of ‘knowing and being known’” [(50), p. 40]. This is the moment of meeting from which the sense of authenticity derives. In the experience of knowing and feeling known, one feels vital, real.

Sander's application of systems theory to moments of meeting is consonant with Aday and Schmader's (69) assertion “that authenticity is a subjective signal of fit to one's environment” and is “more often experienced as a state of mind rather than a trait of the person” (p. 2). They add, “the presence of fit and fluency” between the individual and her/his environment “will lead to a sense of authenticity” (p. 3).

The sense of authenticity derived from implicit relational knowing in psychotherapy is co-constructed through a process of contingent responses in psychotherapy which parallel the way that the True Self, the sense of authenticity, emerges as a potential in the infant-mother dyad. “From birth, the infant is a “contingency detector” [(70), p. 826]; the infant can detect what is and what isn't a contingent response to what is in her mind.

It is no easy task for the mother to know what is in the mind of her infant.

Observation of videotapes of parents and infants during the first year further reveals that the parent actively scaffolds the infant's ability to articulate and communicate his mental states somewhat ahead of the infant's ability to do so himself. Thus, the parent inducts the infant into the role of communicative partner (building on the infant's preadapted ability to participate as a social partner) by responding carefully to infant nonlinguistic initiatives as communications and by taking the infant's turn in conversation until the infant can fill the turn himself, for example, to a 2-month-old: “Does that noise mean you're hungry? Maybe you're hungry. Let's see if you want this water? No? No water? How about juice? Ok, you like that!” [(71), p. 583].

When this process of interactive error and repair, with its alternating miscoordination and coordination of intention [see (72)] eventuates in “Ok, you like that!”, it illustrates Winnicott's experience of simultaneously creating and finding. As the mother is “ahead” of the infant, it is in the finding of juice that the infant finally recognizes the true nature of her vaguely experienced need. The specific nature of the need is created/known by the finding of its object [compare with (39), p. 61, quoted above].

When implicit relational knowing applies to affects, intentions, and motives, rather than physical needs, the negotiation between two subjective systems gives rise to the potential for both greater coherence and complexity, a positive way of being with another that feels authentic and meaningful which emerges from a shared sense of knowing and being known. But when not openly responded to, added complexity also carries the risk of “various kinds of deletions and distortions or ‘incoherencies,”’ [(71), p. 590] which can lead to interpersonal defensive strategies, such as not exhibiting affects to others, basing social interactions on compliance (False Self object relations), or segregating certain experiences “outside the process of ongoing regulation in the parent-child dialogue” [(71), p. 591]. The latter strategy may relegate traumatically-experienced events or interactions to what Bollas (73) termed the “unthought known.” Bollas holds that in the transference, one can “live through for the first time elements of psychic life that have not been previously thought” [(73), p. 278]. The unthought known, therefore, is the repository for that which was never consciously lived because procedurally excluded. In explaining his concept, Bollas “turn(s) quite naturally to Winnicott's concept of the true self to indicate what I believe this previously unlived something is” (p. 278). *Authenticity (the True Self) is brought to life through intersubjective knowing*.

I also suggest that the unthought known may account for the vague but powerful sense of familiarity (déjà vu) one may sometimes have in encountering something for the first time, as well as the sense of confidence expressed in the phrase, “I know it when I see it.”^5^ As discussed further below, confidence based in that which is known implicitly, crystallized through intersubjective moments of meeting, is also evident in the conviction with which psychedelic-based insight is held.

Extrapolating from Lyons-Ruth et al.'s (71) point above, the unthought known may also be a repository for the “*not-me*” (75), traumatic experiences which become disavowed or disassociated as “deletions” from the process of intersubjective attunement. Lyons-Ruth et al. emphasize that attachment research has shown consistently (76) that many defenses which arise from early failures of attunement are not “one person” intrapsychic defenses such as repression which divide conscious from unconscious, but derive from interpersonal strategies that lessen the affective disruption and emotional pain of inconsistent or failed attunement to subjective experience. This has implications for how psychedelics reduce defensiveness (see below). These early arising defensive strategies for minimizing affective arousal are later generalized into severely constrained implicit procedures for being with others (67), which lead to disorganized attachment and interfere with the experience of knowing and being known (77–79). Beebe and colleagues also recognize the connection between knowing and being known in psychoanalytic therapy and feeling “alive” or “real” (77, 80, 81).

In the psychotherapeutic setting, such “moments of meeting” are rare. They are more intense than “present moments” and even “now moments” (which carry ascending levels of authenticity) and involve some clear (to both parties) departure from usual roles. This is essential for a moment of meeting. As Stern et al. (60) put it: “When we speak of an ‘authentic’ meeting, we mean communications that reveal a personal aspect of the self that has been evoked in an affective response to another. In turn, it reveals to the other a personal signature, so as to create a new dyadic state specific to the two participants” (p. 917). From the patient's point of view, an aspect of the “real” therapist (outside of the transference, i.e., the usual way the patient experiences the therapist) is revealed by the therapist in the exchange and ratified as such (62). “Mutual regulation is based on each partner's ability to detect that the partner's behavior is contingent on his own actions, and vice-versa” [(82), p. 37]. It is the emergence within the dyad of this “real” aspect of the analyst, the “personal signature” *as a contingent response to the analysand*'*s experience* that confers the sense of authenticity to the analysand in the moment of meeting. The spontaneous affective reaction of the therapist lets the patient know she is known. Though the moment of meeting is instantaneous and may not be verbalizable, if it were, it might go something like: “I must have surprised you, but in your contingent reaction I see the real way I affect you, unobstructed by your usual restraint. In your genuine reaction to me, I see a new aspect of myself, the me that caused this reaction in you, which, absent your spontaneous response, I would never have known existed.”

This glimpse of the real analyst has immediate implications for the process of relinquishing the omnipotent object, or adapting to the “objectively perceived” object who is not always attuned to what one is experiencing. In the next section, we will examine the way this process is addressed in psychedelic-assisted psychotherapy.

## Psychedelic-assisted psychotherapy and expectancies

Moments of meeting depend upon implicit relational knowing, which develops from pre-linguistic, cross-modally conveyed correspondences, including affect attunement (49, 83). Beebe and Lachmann note, “All linguistic forms of intersubjectivity continue to depend on pre-linguistic forms…. However, pre-linguistic forms of communication can come to the fore when later aspects of development falter [(82), p. 37]. Psychedelics cause later aspects of development– broadly what Freud [(84), p. 588] termed the secondary process– to falter. With high doses,^6^ perception becomes sensitized to vitality affects, the contours of activation, intensity, and timing that shape the way affects are behaviorally expressed, which are the basis for cross-modal correspondences. By enabling contact with this earliest, intuitive form of knowing and being known, psychedelic-assisted psychotherapy engenders a sense of authenticity.

How does psychedelic-assisted psychotherapy fit in with the dynamics of the intersubjective process discussed above? Upon initial inspection, it would seem not to fit in at all, especially in the manner that psychedelic-assisted therapy is conducted in the research setting nowadays, with the subject wearing an eye mask and headphones. Obviously, not seeing the face of the therapist is not very conducive to alterations in the implicit therapeutic relationship that might lead to moments of meeting. Fortunately, the story does not end here.

Ratcliffe (26) suggests “that changes in existential feeling involve changes in a diffuse, background sense of bodily dispositions, which are at the same time changes in the kinds of possibility that the world accommodates. I use the term ‘background’ to emphasize that existential feelings are presupposed by the possibility of intentional states, there in advance” (p. 19). As discussed above, psychedelics induce changes in one's sense of bodily dispositions which alter one's sense of reality. Ratcliffe's statement suggests that another way of construing this altered sense of reality is as a change in the possibility of intentional states one may experience. I suggest that psychedelics alter the possible intentional states one may experience by activating the same dynamics which arise in moments of meeting; that is, the drugs induce a sense of knowing and feeling known by another, which may be a therapist, a representation of a key figure in someone's life, an evoked companion (49), a vague imagined audience, or even a supernatural entity. What unites this range of real and imagined transactions is the experience of being known by an object. It is this experience which confers the sense of authenticity to knowing.

What makes these drugs specifically conducive to this coinciding of knowing and being known is the way they alter expectancies. There are several theories about how MDMA (36) and psychedelics (87) exert their characteristic neurophenomenological effects. Most of them, including the most widely accepted one (11) are based on Friston's (88) free energy principle, which proposes that living organisms are self-evidencing systems which resist the natural physical tendency toward entropy by reducing surprise or uncertainty [see also (89)]. This is achieved by hierarchical predictive processing within a nervous system that generates Bayesian probabilistic models of its environment, and then samples it. By activating layer V pyramidal cell 5HT2A receptors, psychedelics sensitize and dysregulate the highest levels of the brain's hierarchical structure, “subverting the brain's ability to entrain and constrain emotion and perception to a central narrative: “The Centre cannot hold.” (Yeats, 1865–1939)” [(11), p. 325]. This lowers the precision (felt confidence in) models generated by the top, or “Centre” of the predictive processing hierarchy. While the structural location of the center is under debate (10, 13, 90), and even its phenomenological identity (minimal v. narrative self) [see (91)], the view expounded here, based on decades of infant-mother dyad observation applied to psychoanalytic theory beginning with Winnicott and summarized by Beebe (61), is that the higher order generative models most relevant to negotiating the interpersonal world are not models of self, but models of self with others. They are models of interpersonal and intersubjective expectancies.

Temporarily relaxing the precision of intersubjective expectancies means no longer expecting emotional pain from efforts to share subjective experience, thereby obviating the defensive strategies adopted to minimize this pain. Both MDMA (92, 93) and psychedelics (94–96) are reported to reduce defensiveness. Of course, this is not an all or nothing phenomenon, and may be related to dose and a variety of other set and setting conditions. It is maximal in full-blown ego dissolution, which following the argument above, might more accurately be viewed as “self-with-other expectancies dissolution.” Eliminating defenses that protect one from expected pain in attempting to share experience opens the door to knowing and feeling known, and thus contact with the True Self and the sense of authenticity.

## Psychedelic-assisted psychotherapy and implicit others

To be clear, I do not advocate the idea that eye masks and headphones are essential for conducting psychedelic-assisted psychotherapy, especially in some (back to the) future model in which a psychedelic might be used as an occasional adjunct to a longer-term psychodynamic transference-based treatment, in which seeing the therapist's face is a big part of the implicit therapeutic relationship. But to the extent that these devices promote immersion into an inner world, they may actually facilitate communion with imagined presences.^7^ These presences are vital to the process of psychedelic-assisted psychotherapy, and to the process of feeling alive in general.

A brief analogy can help make the point. There is a philosophical thought question that goes: “If a tree were to fall on an uninhabited island, would there be any sound?” One answer is: “Sound is vibration, transmitted to our senses through the mechanism of the ear, and recognized as sound only at our nerve centers. The falling of the tree or any other disturbance will produce vibration of the air. If there be no ears to hear, there will be no sound” (97). Likewise, if someone has an experience, and no one is around to tell it to, did it really happen? Without getting lost in philosophical debate, one may say that subjectively, the sense of reality associated with the experience is far greater when the subject senses a witness to the experience. This holds true even if the witness is only imagined.

Donnel Stern's extraordinarily insightful paper on “Partners in Thought” (63) gets right to the heart of the matter. “We need to feel that we exist in the other's mind and that our existence has a kind of continuity in that mind; and we need to feel that the other in whose mind we exist is emotionally responsive to us, that he or she cares about what we experience and how we feel about it” (p. 706). Witnesses may be constructed in a variety of ways. “The witness, while it may feel like a single presence, may nevertheless be composed of part(s) of one's own mind or of the other's, or of both simultaneously. The witness is the state(s) of self and/or other who one imagines is best suited to fulfill the partnering purpose at the particular moment in which the need arises” (p. 707). Stern's point is that in order to know our own story, our “self,” we need to experience that story as being heard by another. Absent an actual or imagined listener, our experience lacks real meaning. “Without a witness, even an imaginary one, events either fail to fall into the meaningful pattern of episode that is narrative, or we merely enact our stories blindly, unable to think about them or know what they feel like. Our witness is our partner in thought” (p. 706).

In MDMA-assisted psychotherapy for PTSD, the source of healing is sometimes described as an “inner healer” or a “native healing intelligence” (98) I suggest this somewhat vague term can be better understood in Stern's conception, as a partner in thought. Without one's customary defenses against being misunderstood, unrecognized, it is easier to have the kind of inner dialog Stern describes. And in the case of ego dissolution, when self-as-subject (the experiencing “I”) is detached from self-as-object, new forms of this dialog may take place.

Stern compares telling one's story to an imagined audience to the way toddlers babble to themselves animatedly after being put to bed. This is a time when transitional phenomena are often engaged. To whom exactly is the child talking? At times it is possible to detect in the cadence and intonation of the child's voice an impression of another, such as a parent. In such a case, it may be understood that the child is identified with a parent who is talking to the child. This is a very interesting transitional phenomenon. In Winnicott's formulation of transitional phenomena, the child's subjectivity occupies the potential space between subject and object. In the instance of babbling, the notion of seeing oneself reflected in the face of the other takes on new meaning. *The child*'*s center of subjectivity, for the moment, resides in the object who is relating to the child*, though undoubtedly it soon re-centers within the subject/child. This *fluid transfer of the center of subjectivity between partners in thought* may be one of the mechanisms through which we see ourselves reflected in, or feel known through the object, even in the object's physical absence. Where the distinction between subject and object is fluid or blurred, so is the distinction between knowing and being known. When what one “knows” feels simultaneously “known” by a trusted other, it feels ratified, real.

I suggest that the noetic sense which attaches to insight in psychedelic-assisted psychotherapy derives from this coinciding of knowing and feeling known. In this context, knowing and feeling known reflects a generalizable process, a way of belonging in the world, which arises from a unique moment of meeting. That is, it is an existential feeling in Ratcliffe's sense that is amplified by the drug. Stern: “The witness begins as that kind of internalization [of a specific object], but becomes a changing amalgam of history, fantasy, and current reality. It is not a structure of the mind, but a function—or, better, a way of being” (p. 707). This “way of being,” of being known and thus transformed by the object, aligns with Bollas's (99) notion of the transformational object as *a process of change*.

When the issue at hand is precisely the consequence of not feeling known by a trusted other, when the relinquishing of the omnipotent object has been fraught with inconsistency, mis-attunement, or repeated trauma, psychedelic-assisted psychotherapy enables one to engage the process of illusion-disillusionment with respect to the object. The relaxed precision of generative self-with-other models, or in other language, the self-as-subject's disconnection from self-as-object of trauma enables the experiencing of this object in a new light, absent the defensive distortions and intolerable affect normally associated with the trauma-causing object. Such experiences enable a re-calibration of one's perception of the object, reduce its omnipotence (i.e., its power over the subject*)*, place it in a more objectively perceived context. As an unfettered transitional space, psychedelic-assisted psychotherapy engages the creative imagining of other ways of being with the frustrating or traumatizing object.

Psychedelic-assisted psychotherapy, then, is a process where the distinction between knowing and being known collapses as a result of experiencing oneself as one's partner in thought, or experiencing oneself as existing in a transitional space between subject and object. The setting itself, the over-arching presence of the therapist as a “partner in thought,” enriches this experience, even with an eye mask in place. Certainly, this resonance is deeper when trust has been burnished through long-term therapy.

## Final comments

The model of knowing and being known discussed above allows adaptation to the specific need and moment in which it arises. The nature of the witness, and the nature of what parts of oneself, and even whom is witnessed, is malleable and changes according to need and context. Moments of meeting alter ways of being with others; both subject and object are altered by the meeting of minds. As Stern (63) writes: “It is not only the witness who is in flux, however; the one who is witnessed is as well, since the state of self in need of witnessing also changes with context” (p. 707).

Psychedelic-assisted psychotherapy, in removing the constraints imposed by canalized ways of being with others, invites creative re-imagining of ways of knowing and being known. Its effectiveness in bringing to bear the process of knowing and being known in a transitional space upon that which was experienced with some combination of “awe, horror, loathing, or dread”– Sullivan's “*not-me*”—[(75), p. 163], may explain its effectiveness in helping to “facilitate recall of negative or threatening memories with greater self-compassion and less PTSD-related shame and anger” [(100), p. 1032].

Winnicott implores us to accept the paradox that the subject both creates and finds the object which satisfies its need. In accepting the paradox, we agree not to ask the subject whether she/he created or found the object. Following from the discussion above, in evoking a witness, the subject both creates and finds the object which satisfies his/her need to know and be known. This allows us to express the paradox differently: *In creating the object, the subject is found*. In finding ourselves in a knowing object, we feel a sense of authenticity, we create meaning which may “carry… a curious sense of authority for after—time.”

## Data availability statement

The original contributions presented in the study are included in the article/supplementary material, further inquiries can be directed to the corresponding author.

## Author contributions

The author confirms being the sole contributor of this work and has approved it for publication.

## Conflict of interest

The author declares that the research was conducted in the absence of any commercial or financial relationships that could be construed as a potential conflict of interest.

## Publisher's note

All claims expressed in this article are solely those of the authors and do not necessarily represent those of their affiliated organizations, or those of the publisher, the editors and the reviewers. Any product that may be evaluated in this article, or claim that may be made by its manufacturer, is not guaranteed or endorsed by the publisher.

## Footnotes

^1^While some of these studies suggest that such changes may be more pronounced with “classic” psychedelics than with MDMA, I have included studies here which report changes in bodily experience with MDMA, as I am less concerned with degrees of differences than with the way both change the pre-reflective way that one experiences the world.

^2^Winnicott also views the True Self as a “potential” (see above).

^3^Though I no longer accept as verbatim Winnicott's formulation about the infant's “omnipotence,” I leave intact above my initial impression of it to illustrate the process. I attribute below to psychedelic-assisted therapy, which inactivates working models of being with others (e.g. compliance with authority) and enables the experience of authenticity. By eventually relinquishing my compliance with Winnicott's “authority,” I constructed what feels like a more authentic model of transitional phenomena and knowing and being known.

^4^“The Place where we Live” (51) is an aptly-titled chapter in *Playing and Reality* (37) in which Winnicott elaborates ideas he also addressed in a previous chapter, “The Location of Cultural Experience” (45). The theme of the two chapters is that it is not instinctual gratification “that makes a baby begin to be, to feel that life is real, to find life worth living” [(45), p. 98], but the experiences of playing and using the elements of cultural heritage in the transitional space between psychic reality and external reality.

^5^This line was famously used by Justice Potter Stewart in Jacobellis v. Ohio 378 U.S. 184, 197 (1964), a case about whether a lovemaking scene in a Louis Malle movie, *The Lovers*, constituted pornography: “But I know it when I see it, and the motion picture involved in this case is not that.” Paul Gewirtz (74), writing about Stewart's use of this line, opined, “Indeed, there is something familiar about both its rhetoric and content, its symmetrical equation of seeing and knowing, and its insistence that some knowledge comes immediately from seeing, not from deliberating” (p. 1024). I suggest this intuition has its roots in implicit relational knowing.

^6^Psychedelics in high doses, moreso than MDMA, can cause synesthesia, perhaps the clearest example of cross-modal convergence, but the point is that these drugs incline perception towards the primary process, *in the direction of “coenesthetic experience”* (49, 85, 86) even if synesthesia is not experienced directly.

^7^There is suggestive parallel between the adopted custom of wearing of eyeshades in psychedelic-assisted psychotherapy and the use of the couch in Freudian psychoanalysis, in keeping the subject/analysand from seeing the face of the psychotherapist.
